# Frequency of HIV status disclosure, associated factors and outcomes among HIV positive pregnant women at Mbarara Regional Referral Hospital, southwestern Uganda

**DOI:** 10.11604/pamj.2019.32.200.12541

**Published:** 2019-04-24

**Authors:** Joseph Ngonzi, Godfrey Mugyenyi, Mukasa Kivunike, Julius Mugisha, Wasswa Salongo, Sezalio Masembe, Ronald Mayanja, Francis Bajunirwe

**Affiliations:** 1 Mbarara University of Science and Technology, Department of Obstetrics and Gynecology, Mbarara, Uganda; 2 Mbarara University of Science and Technology, Department of Community Health, Uganda

**Keywords:** Disclosure, HIV/AIDS, Mbarara University, Mbarara hospital, Uganda, factors associated

## Abstract

**Introduction:**

Positive HIV results disclosure plays a significant role in the successful prevention and care of HIV infected patients. It provides significant social and health benefits to the individual and the community. Non-disclosure is one of the contextual factors driving the HIV epidemic in Uganda. Study objectives: to determine the frequency of HIV disclosure, associated factors and disclosure outcomes among HIV positive pregnant women at Mbarara Hospital, southwestern Uganda.

**Methods:**

A cross-sectional study using quantitative and qualitative methods among a group of HIV positive pregnant women attending antenatal clinic was done and consecutive sampling conducted.

**Results:**

The total participant recruitment was 103, of which 88 (85.4%) had disclosed their serostatus with 57% disclosure to their partners. About 80% had disclosed within less than 2 months of testing HIV positive. Reasons for disclosure included their partners having disclosed to them (27.3%), caring partners (27.3%) and encouragement by health workers (25.0%). Following disclosure, 74%) were comforted and 6.8% were verbally abused. Reasons for non-disclosure were fear of abandonment (33.3%), being beaten (33.3%) and loss of financial and emotional support (13.3%). The factors associated with disclosure were age 26-35 years (OR 3.9, 95% CI 1.03-15.16), primary education (OR 3.53, 95%CI 1.10-11.307) and urban dwelling (OR 4.22, 95% CI 1.27-14.01).

**Conclusion:**

Participants disclosed mainly to their partners and were comforted and many of them were encouraged by the health workers. There is need to optimize disclosure merits to enable increased participation in treatment and support programs.

## Introduction

HIV/AIDS remains a major public health problem and more effort is needed to ensure successful treatment and prevention programs. Disclosure is an important component in uptake of prevention of mother-to-child transmission (PMTCT) services [1]. Women are counseled to share with their partner their own HIV test result and they become responsible for encouraging their partner to undertake HIV testing. The dialogue on sexual activity or HIV/AIDS within a couple is often difficult, especially when women discover that they are HIV-infected [2]. Among postpartum mothers, disclosure is associated with adherence to safer infant feeding practices, exclusive breast feeding, exclusive replacement feeding, creates awareness about HIV risk for the untested sexual partners, supports risk reduction, promotes safer sexual behavior, increased retention in eMTCT programs, leads to better clinical outcomes such as CD4+ count increases and higher rates of retention in treatment programs [3-12]. Studies also show that disclosure is a potential strategy for dealing with stigma among patients receiving antiretroviral therapy [13]. Despite these potential benefits, studies indicate the frequency of non-disclosure remains relatively high. About 25% of HIV infected patients do not disclose their HIV serostatus to their partners [14]. The proportion of women disclosing their HIV serostatus to their partners among HIV positive pregnant women attending antenatal care is even larger (60%) [15]. Disclosure is a complex process that requires delicate handling to prevent occurrence of unwanted effects [16]. HIV status disclosure can be a period of heightened risk for partner stigma, abuse and financial withdrawal and thus should be handled with caution [17, 18]. Therefore, the purpose of this study was to determine the frequency, factors associated with disclosure and the potential barriers and facilitators to disclosure of HIV serostatus by pregnant women attending antenatal clinic at Mbarara Regional Referral Hospital (MRRH), South Western Uganda.

## Methods

**Study site:** The study was conducted at Mbarara Regional Referral Hospital Antenatal Care clinic (ANC). The study was conducted among HIV positive pregnant women attending the antenatal clinic at Mbarara Regional Referral Hospital ANC. The antenatal clinic sees 700 women per month at least 90% of women in Uganda attend at least one antenatal visit during their pregnancy. The antenatal HIV prevalence in Uganda is 6.4% while the prevalence in Mbarara Hospital maternity ward is about 12% [19]. Mbarara Hospital is both a teaching hospital for Mbarara University Medical School and Regional Referral Hospital located within Mbarara Municipality, Mbarara district in the Western region of Uganda about 270Km from the capital city Kampala. The facility provides care for a diverse ethnic group of patients from the region, including some parts of the Democratic Republic of Congo, Rwanda and the Northern part of Tanzania.

**Study design:** The study was a cross sectional study using both quantitative and qualitative methods. The quantitative method involved use of interviewer-administered pre-tested questionnaires while the qualitative method involved two focus group discussions (FGDs) i.e. eight HIV positive women who had disclosed their serostatus and eight positive women who had not disclosed.

**Sample size and sampling procedure:** The sample size calculated was 103 women using the formula by Kish and Leslie (1965). The sample size was calculated based on the assumption that 20% of women attending antenatal clinic will not disclose HIV results to anyone. Using an error margin of 7.5%, an estimated 103 women were sufficient to answer the study objectives. The HIV positive pregnant women attending MRRH ANC were consecutively recruited until the desired number of 103 was achieved while every 5^th^ respondent during the quantitative survey was also requested to participate in a focus group discussion until the required number of eight participants per group.

**Data collection and instruments:** The HIV positive mothers were received at the registration desk with the rest of the pregnant women. They were identified using the HIV status codes on their ANC charts. The clinician handed them over to a research assistant in a separate room away from the routine antenatal clinic activities. The research assistant provided the mothers with information about the study and sought their consent to participate. Quantitative data was collected using interviewer-administered pre-coded pre-tested questionnaires to determine: The socio-demographic variables such as age, marital status, residence, employment, education level, nature of domicile, religion, parity and tribe. The disclosure status (disclosed or not disclosed), to whom, when it was done, and the outcomes of disclosure were collected. The questionnaire was pre-tested and translation double verified. The questionnaire was piloted, and necessary adjustments made. The data was cleaned. During the focus group discussions, the interview was recorded using a voice recorder.

**Data entry and analysis:** Quantitative data was entered into the EPI-INFO program and analyzed using the statistical package for social science (SPSS version 12). The categorical data was summarized into frequencies or proportions. The socio-demographic characteristics of women who disclosed their HIV serostatus were analyzed. The primary outcome for the quantitative analysis was disclosure. The association between social, economic and demographic categorical variables with disclosure status was obtained using binary logistic regression analysis and an association was considered significant if the p-value was less than 0.05. The qualitative data was verbatim transcripted, categories created with evidences from the responses and coded using the thematic content analysis.

**Ethical consideration:** The work was presented to the department of obstetrics and Gynecology at Mbarara University and ethical approval sought from the faculty research ethical committee. Individual consent was obtained from the participants enrolled. Only participants who voluntarily agreed to participate in the study received an interview.

## Results

Majority of the participants were between the ages of 18 and 35 years (94.2%), Christians (89.3%), had primary education (56.3%), were married either monogamously or polygamously (94.1%), were multipara (61.2%), lived in a nuclear family setting (71.8%), unemployed (59.2%) and had a monthly income of between 50,000-100,000 Uganda shillings (42.7%). Among the respondents who were below 18 years, 83.3% had disclosed their serostatus. Disclosed among those who had no formal education was 100% (Table 1). Persons disclosed to included partners (57%), parents (25%), friends (9%), relatives (6%) and siblings (3%). Out of the 103 respondents, 88 (85.4%) had disclosed to at least someone. About seventy nine percent (79%) had disclosed within less than 2 months of testing positive while 9.1% had disclosed after 6 or more months of having tested positive (Table 2). One of the respondents in the focus group discussions reported having disclosed within seven days as evidenced by her response i.e. *"I told my husband on the second day following my testing positive because he had disclosed to me his serostatus and was openly taking his HIV drugs"* said 30-year-old mother of three. Most women who disclosed their sero-status were encouraged by health care workers, had partners who were caring and had disclosed to them (Table 3). This was further supported by information gathered from the focus group discussions where participants reportedly disclosed because their partners had disclosed to them and some had been encouraged to do so by the health workers as said by participants a 32-year mother of four children and 22 year a primipara respectively: *"I told my husband on the second day following my testing positive because he had disclosed to me his serostatus and was openly taking his HIV drugs". “The health worker always reminded and encouraged me whenever we met and I got the boldness to tell my husband ”*

**Table 1:** The socio-demographic characteristics in relation to disclosure

Variables	Non-disclosure n=15 (%)	Disclosed n=88 (%)	P-value
**Age**			
Below 18	1(6.7)	5 (5.6%)	0.40
26-35	6 (40)	43 (48.9%)	0.05
18-25	8 (53.3)	40 (45.5%)	
**Religion**			
Catholic	6 (40)	32 (36.4%)	
Anglican	7 (47)	44 (50%)	0.20
Others	2 (6.5)	12 (12.5%)	0.64
**Education level**			
No formal or Primary	10 (66.7)	58 (66%)	0.03
Post-primary	5 (33.3)	30 (33%)	
**Marital Status**			
Married monogamously	9 (60)	57 (64.8%)	0.26
Married polygamously	3 (20)	27 (30.7%)	0.51
Others (Single/Separated)	3 (20)	4 (4.5%)	
**Parity**			
Prime gravida	5 (33.3)	26 (29.5%)	
Multipara (2-4)	9 (60)	55 (62.5%)	0.41
Grand multipara (5 or more)	1 (6.7)	7 (8%)	0.65
**Domicile**			
Nuclear family	74 (71.8)	62 (70.5%)	0.45
Extended family	29 (28.2)	26 (29.5%)	
Residence			
Urban	10 (66.7)	44 (50%)	0.02
Rural	5 (33.3)	44 (50%)	
**Patient employment**			
Unemployed	9 (60)	53 (60.2%)	0.83
Informal sector	4 26.7)	29 (33%)	0.38
Formal/skilled	2 (13.3)	6 (6.8%)	
**Monthly income (Uganda shillings)**			
Less than 50,000	4 (26.6)	25 (28.4%)	
50,000-100,000	7 (46.7)	39 (44.3%)	0.53
More than 100,000	4 (26.7)	24 (27.3%)	0.76

**Table 2:** Frequency and timing of disclosure among women attending antenatal clinic at Mbarara Regional Referral Hospital

Variable	Frequency (percent %) N (%)
**Disclosure status**	
Yes	88 (85.4)
No	15 (14.6)
**Disclosure timing**	
Less than 2months	70 (79.5)
2-5months	10 (11.4)
6 or more months	8 (9.1)

**Table 3:** Factors that motivated disclosure among pregnant women who disclosed their HIV sero-status, Mbarara Hospital, Uganda (n = 88)

Factors motivating disclosure	Frequency (percent %)
He had disclosed to me	24 (27.3)
He was caring	24 (27.3)
I was financially stable	7 (8.0)
Wanted safer sex	11 (12.5)
Encouraged by health worker	22 (25.0)

**Reasons for non-disclosure:** included fear of abandonment (32%), being beaten (32%), loss of financial support (12%), stigmatization (12%), loss of emotional support (6.7%) and others thought that disclosure was not necessary (6.7%). The above information from the questionnaires was supported by information gathered from the focus group discussions where women reported fear of death, divorce, being beaten, job denial and ignorance of the importance of disclosure were reported as some of the barriers to disclosure. Some of the responses included the following i.e. *"Knowing that he easily gets upset by small things and begins fighting, if I tell him about my status will he not beat me to death? His brother beat his wife seriously when he found that she was positive, and he is now in prison"* said 29-year old primary school teacher. *"Can't I live with my disease without bothering people by telling them of my issues? In feel comfortable that way"* reported a 40-year-old prisons warder and a mother of 6 children. *"I am looking for a job right now and if probable employers get to know that am positive, they may deny me a job. I will reveal my status when I have a job"* reported a 34-year-old mother of 3 children.

**Post disclosure experiences:** Majority of the women who disclosed were comforted (73.9%). However, negative outcomes included accusation of infidelity (24.9%), others were verbally abused (6.8%), some were beaten (5.7%) and a few were chased out of their homes by their husbands and the relatives of their husbands (2.3%). The information from the focus group discussions lent further credence to that gathered from the questionnaires with respondents reporting increased support and comforting as said by a 25-year old mother of 3: *"When I told my mother that I was HIV positive, she was so sad but later comforted me and promised to give me all the support I needed".* She had also told her partner *"My partner pledged his support and continued love till death do us part. He has always reminded me to take my drugs and goes with me to hospital during my clinic days".* Negative, some mothers reported financial loss, being beaten, denial of conjugal rights, divorce and stigma as some of the outcomes of their having disclosed their serostatus. Some of their responses included the following i.e. *"When I told my partner, he beat me that night and locked me in the house for two days though he came back to his senses and stopped harassing me"* reported 18-year-old primipara.

**Factors associated with disclosure:** The factors associated with disclosure were age between 26-35 years. This age group has 3.9-fold increase in the odds of disclosure compared to those in the 18-25 year age category (OR 3.9, 95% CI 1.03-15.16). Primary education was associated with a 3.5-fold increase in the odds of disclosure (OR 3.53, 95% CI 1.10-11.307) compared to those with post primary education. Urban dwelling was associated with an over 4-fold increase in the odds of disclosure compared to the rural folk (OR 4.22, 95% CI 1.27-14.01) (Table 4).

**Table 4:** Factors associated with disclosure of HIV among pregnant women attending antenatal clinic at Mbarara Regional Referral Hospital

Variable	Odds Ratio (95%CI)	P-value
**Age**		
Less than 18	2.9 (0.25-33.07)	0.40
26-35	3.9 (1.03-15.16)	0.05
18-25	1	
**Education**		
No formal or Primary education	3.53 (1.10-11.307)	0.03
Post primary	1	
**Residence**		
Urban	4.22 (1.27-14.01)	0.02
Rural	1	

## Discussion

About 85% of the participants had disclosed their HIV serostatus to at least one person and of these participants, 57% had disclosed to their partners. Almost 80% had disclosed within less than 2 months of testing HIV positive. Women disclosed their serostatus because their partners had disclosed to them (27.3%), their partners were caring (27.3%) and health workers had encouraged them to disclose (25.0%). Following disclosure, majority were comforted (73.9%) while others (6.8 %) were verbally abused. Those who did not disclose feared abandonment (33.3%), being beaten (33.3%) and loss of financial and emotional support (13.3%). The factors associated with disclosure included age group 26-35 years, primary education level and urban residence. A study in Dar Es salaam, Tanzania in an ANC clinic interviewing HIV positive women about disclosure to their partners found that 69% had disclosed to their partners [20]. The overall HIV status disclosure to sexual partner in a study in Ethiopia was 57.4% and the study showed that there is significant association between knowing HIV status of the sexual partner [21]. The rate of disclosure to partners in our study was 57%. These rates of disclosure to partners are almost similar probably because the settings were almost the same and the populations studied were from the low resource settings and had similar socio-economic and demographic characteristics.

**Negative disclosure outcomes and reasons for non-disclosure:** in our study, women who disclosed were accused of infidelity, others were verbally abused, beaten, and a few were chased out their homes by their husbands and the relatives of their husbands. The women feared abandonment, being beaten, loss of financial support, stigmatization, loss of emotional support and others did not know the importance of disclosure. The three commonest reasons for non-disclosure in other studies are fear regarding spread of the information, stigmatization and deterioration in the relationship with the spouse [22]. This could be because this group of women considered the disclosure process to be too difficult and risky to undertake and engaged in avoidant behaviors to hide their HIV status. Women who do not disclose their HIV status to their sexual partners sometimes do not practice safer sex, especially condom use and it is possible that this group of women may be more likely to have re-infection [23]. The negative outcomes may lead women to choose not to share their HIV test results with their friends, family and sexual partners. This, in turn, leads to lost opportunities for the prevention of new infections and for the ability of these women to access appropriate treatment, care and support services where they are available.

**Positive disclosure outcomes:** Most women who disclosed their HIV serostatus in our study were comforted and now able to participate in HIV treatment programs. Disclosure of HIV status expands the awareness of HIV risk to untested partners, which can lead to greater uptake of voluntary HIV testing and counseling and changes in HIV risk behaviors. In addition, disclosure of HIV status to sexual partners enables couples to make informed reproductive health choices that may ultimately lower the number of unintended pregnancies among HIV-positive women [9]. Among women, who disclose their HIV serostatus to their families, friends and sex partners, the incidence of regret is minimal and that disclosure improved on relationship satisfaction and security [24]. Disclosure is necessary to initiate discussions about HIV/AIDS and this raises each partner's awareness of the risk of infection and may ultimately lead to behavior change to reduce risk reduction. Disclosure can be an important starting point for HIV positive women to begin discussing the use of contraception with their partners and reduce the number of unintended pregnancies among HIV infected women. Disclosure helps in women's uptake of PMTCT programs and in their participation in treatment and support programs. To benefit from interventions that can reduce HIV perinatal transmission, women who are HIV infected must be willing to accept and adhere to PMTCT prophylaxis. The optimal uptake and adherence to PMTCT programs is difficult for women whose partners are either unaware or not supportive of their participation. It is well documented in Africa that women often lack the power to make independent decisions about their own health care. It is therefore difficult for HIV infected women to seek social and medical support from care and treatment programs for themselves and their infants without first disclosing their HIV serostatus to their partners [25].

**Factors associated with disclosure:** In our study, the reasons given by the women for disclosure were partner having disclosed their status first, partner being caring, encouragement by the health worker and because they wanted to practice safer sex. The factors associated with disclosure included being an urban dweller and having age more than 25 years. This may be because of easy access to information and treatment opportunities compared to the people living in the rural areas. This could also be because those who are older than 25 years are more likely to have spent a longer time in relationships and thus built trust over time resulting into a higher chance to have disclosed compared to the younger ones. The older women are more likely to have gotten pregnant more times than those younger than 26 years and this could have exposed them to more information about disclosure leading to their being more likely to disclose. Disclosure is associated with being married, increased condom use, knowledge of partner's HIV serostatus, knowledge of the partner's status, late stage, staying together with partner, discussion about HIV testing before going for testing, having secondary education, age of more than 25 years, attending more antenatal care visits [26, 27]. Other factors associated with disclosure include having communication skills to disclose, having initiated anti-retroviral therapy, receiving ongoing counselling, having ever seen an HIV infected person publicly disclose their HIV status, being married, knowing the importance of HIV serostatus disclosure and being employed [28, 29]. The strength of our study is that it employed mixed methods to determine responses to multiple aspects of the disclosure are among a vulnerable group of women. The weakness to our study is that it had a small sample size.

## Conclusion

There is heightened need to emphasize the importance of disclosure to enable increased participation in treatment and support programs; and find ways of minimizing the negative consequences and optimizing the positive outcomes of disclosure of the HIV status.

### What is known about this topic

- That disclosure is a potential strategy for dealing with stigma among patients receiving antiretroviral therapy;
- HIV status disclosure can be a period of heightened risk for partner stigma, abuse and financial withdrawal and thus should be handled with caution;
- Disclosure is a complex process that requires delicate handling to prevent occurrence of unwanted effects.

### What this study adds

- The disclosure proportions among HIV-infected pregnant women in Mbarara Hospital;
- The factors associated with disclosure among HIV-infected pregnant women in Mbarara Hospital;
- The reasons for non-disclosure among HIV-infected pregnant women at Mbarara Hospital.

## Competing interests

The authors declare no competing interests.
